# Endoscopic characteristics and high-risk background mucosa factors of early gastric cancer after helicobacter pylori eradication: a single-center retrospective study

**DOI:** 10.3389/fonc.2023.1272187

**Published:** 2023-10-02

**Authors:** Yali Wei, Congcong Min, Chongguang Zhao, Yubei Li, Xiaowei Wang, Xue Jing, Yanan Yu, Xiaoyu Li, Xiaoyan Yin

**Affiliations:** ^1^ Department of Gastroenterology, the Affiliated Hospital of Qingdao University, Qingdao, Shandong, China; ^2^ Department of Clinical Medicine, Qingdao University Medical College, Qingdao, Shandong, China

**Keywords:** *Helicobacter pylori*, eradication treatment, early gastric cancer, characteristics, background mucosa factors

## Abstract

**Purpose:**

Gastric cancer still develops after successful Helicobacter pylori(Hp)eradication. In this study, we aimed to explore the characteristics and risks of mucosal factors.

**Methods:**

A total of 139 early gastric cancers (EGC) diagnosed in 133 patients after successful eradication from January 2016 to December 2021 were retrospectively included in the Hp-eradication EGC group and 170 EGCs diagnosed in 158 patients were included in the Hp-positive EGC group. We analyzed the clinical, pathological, and endoscopic characteristics between the two groups to identify the features of EGC after Hp eradication. Another 107 patients with no EGC after Hp eradication were enrolled in a Hp-eradication non-EGC group. The background mucosal factors between the Hp-eradication EGC group and the Hp-eradication non-EGC group were compared to analyze the high-risk background mucosal factors of EGC after eradication. In addition, we divided the EGC group after Hp eradication into IIc type and non-IIc type according to endoscopic gross classification to assess the high-risk background factors of IIc-type EGC after Hp eradication.

**Results:**

The endoscopic features of EGC after Hp eradication included location in the lower part of the stomach (*p*=0.001), yellowish color (*p*= 0.031), and smaller size (*p*=0.001). The moderate/severe gastric atrophy (GA), intestinal metaplasia (IM) in the corpus, severe diffuse redness, and map-like redness were risk factors for EGC after eradication (*p*=0.001, *p*=0.001, *p*=0.001, and *p*= 0.005, respectively). The Kyoto classification total score in the EGC group was higher than the non-EGC group (4 vs.3 *p*<0.001). A multivariate analysis revealed that depressed erosion (OR=3.42, 95% CI 1.35-8.65, *p*= 0.009) was an independent risk factor for IIc-type EGC after Hp eradication.

**Conclusion:**

EGC after eradication are smaller and yellowish lesions located in the lower part of the stomach. The risk background mucosal factors include moderate/severe GA, IM in the corpus, severe diffuse redness, and map-like redness. The Kyoto classification total score of 4 or more after successful eradication treatment might indicate EGC risk. In addition, the IIc-type EGC should be cautioned in the presence of depressed erosion after Hp eradication.

## 1 Introduction

Gastric cancer is currently the fifth type of cancer in terms of incidence and fourth in mortality disease globally (1), with an especially high incidence in Eastern Asia and Eastern Europe (2). Helicobacter pylori (Hp) plays a key role in the development of gastric cancer (3), by inducing ongoing chronic inflammation, development of gastric atrophy (GA), formation of intestinal metaplasia (IM), and, finally, dysplasia by colonizing the gastric mucosa (4, 5). However, the optimal timing for Hp eradication, which patients are suitable for eradication therapy, and which patients require follow-up after eradication are questions that have been plaguing clinicians. Therefore, more than 40 experts gathered in Kyoto, Japan in 2014 to hold a global meeting and vote on relevant issues. A consensus was finally reached to form the Kyoto Global Consensus Report on Helicobacter pylori gastritis (6). The Kyoto Consensus on gastritis specifically emphasized that patients with Hp infection should receive eradication therapy, which promoted the formation of the global eradication boom. Data from China reported that there would be approximately 509,421 people newly diagnosed and 400,415 people dying from gastric cancer in 2022 in the country, compared with the previous incidence and mortality rates, which represents a gradual decrease (7). This change may be related to the promotion of the Kyoto gastritis consensus in China strengthening the screening and treatment of Hp.

However, with the popularity of eradication therapy, gastric cancer after eradication was gradually discovered and valued. Early gastric cancer (EGC) after eradication was defined as EGC detected after more than 1 year of successful Hp eradication, which contains EGC developing before and after eradication. The former was defined as EGC occurring before eradication but detected after eradication, while the latter was a new cancer occurring after eradication (8). The published data reports that the highest incidence of gastric cancer after eradication can reach 7% (9), and the risk of gastric cancer can persist for approximately 10 years after eradication treatment (10). Therefore, the risk of progression to gastric cancer still exists after eradication. Endoscopists familiar with the endoscopic features and patients undergoing regular endoscopic surveillance may be the key to early detection and treatment of EGC after Hp eradication.

The characteristics of EGC after eradication include small and reddish lesions, flat or depressed morphology, “gastritis-like” appearance under endoscopy, surface differentiation, and non-tumorous epithelium in histology (11–14). Hp eradication can significantly improve diffuse redness (DR), mucosal edema, and enlarged folds (EF), but the gastric mucosa with severe GA and IM in the corpus may be difficult to reverse (15). Meanwhile, eradication therapy improved mucosal inflammation in non-GA and non-IM, making the relatively red IM/AG areas form a characteristic appearance featuring a map-like redness that was in fact multiple slightly flat or depressed erythematous lesions (16). These depressed characteristics and complex background mucosa after eradication greatly increased the difficulty of diagnosis for endoscopists.

This study aims to further explore the characteristics and high-risk background mucosal factors of EGC after eradication. The findings of this study will be beneficial to systematically understand the characteristics and background mucosa of EGC after eradication, and improve the endoscopic detection rate of EGC after eradication.

## 2 Materials and methods

### 2.1 Patients

**Figure 1:**
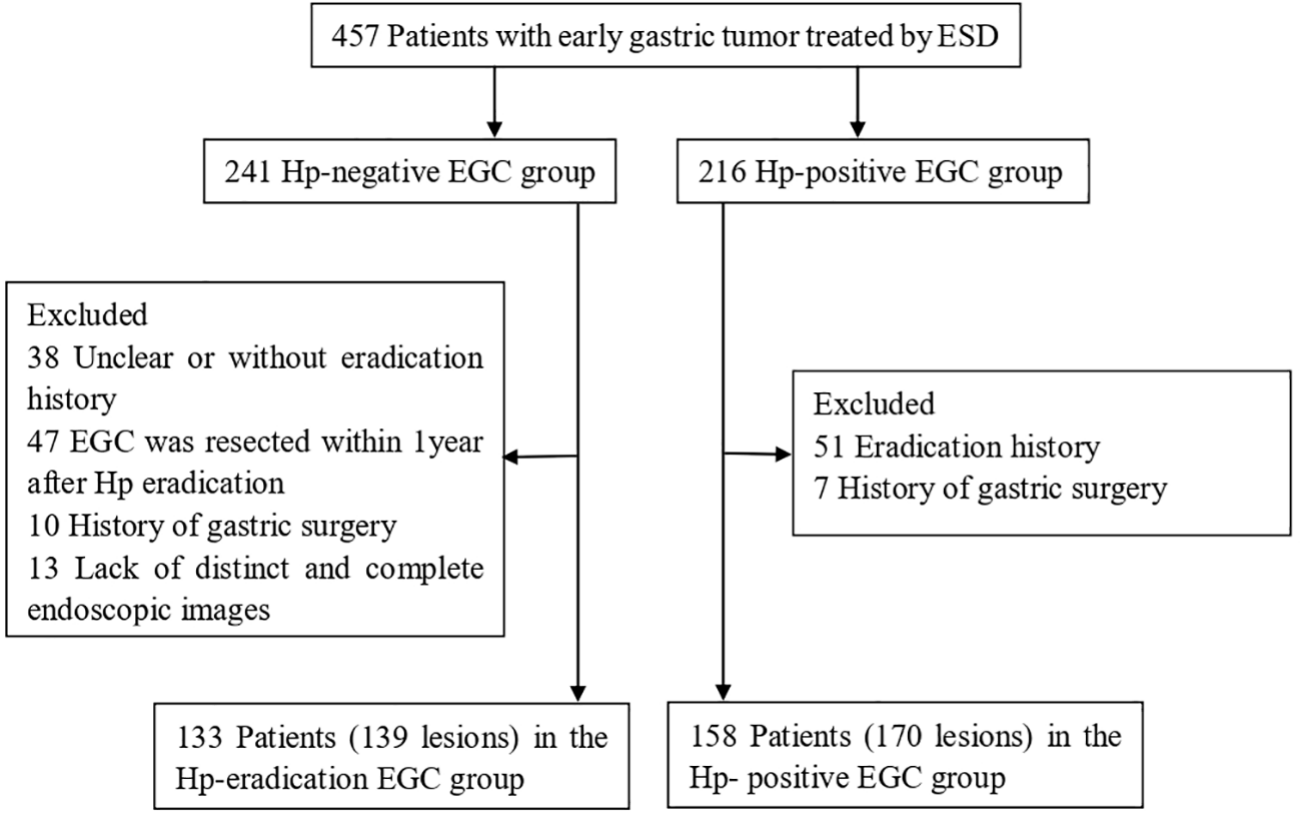
Flowchart of the study subjects.

In this study, 457 patients treated with ESD for early gastric tumors including low-grade, high-grade neoplasia, and early carcinoma in Qingdao University Affiliated Hospital were retrospectively selected between January 2016 and December 2021. According to their pathological results of ESD, the patients were divided into a Hp-negative group (241) and a Hp-positive EGC (216) group. In the former group, 38 patients without or with undefined eradication history, 10 patients undergoing gastric surgery, and 47 patients diagnosed less than 12 months after eradication were excluded. In addition, 13 patients were also excluded for missing or indistinct endoscopic images in the Hp-negative group. In the latter group, 51 patients who had received eradication therapy and 7 patients undergoing gastric surgery were excluded. Finally, 139 lesions in 133 patients who had received successful Hp eradication more than 1 year previously were included in the Hp-eradication EGC group and 170 lesions in 158 patients were included in the Hp-positive EGC group (**Figure 1**).

For the non-EGC group after eradication, 107 patients were randomly selected who met Hp eradication criteria but without EGC for gastroscopy examination at Qingdao University Affiliated Hospital from June 2021 to December 2021.

### 2.2 Standard of successful Hp eradication

After eradication therapy, the eradication effect was evaluated based on the urea breath test or the pathological assessment. The standard of successful Hp eradication was confirmed when either of the tests yielded negative results.

### 2.3 The data at enrollment

Endoscopic characteristics and background mucosa were independently observed by three experienced endoscopists without knowing about the Hp eradication and pathological data, and we finally selected consistent endoscopic results from at least two endoscopists.

### 2.4 The clinical data

The clinical data included sex, age, family history of gastric cancer, smoking and drinking history, and duration after Hp eradication, which was defined as the interval between the successful eradication treatment and the operation of ESD in the Hp-eradication EGC group.

### 2.5 The endoscopic data

**Figure 2:**
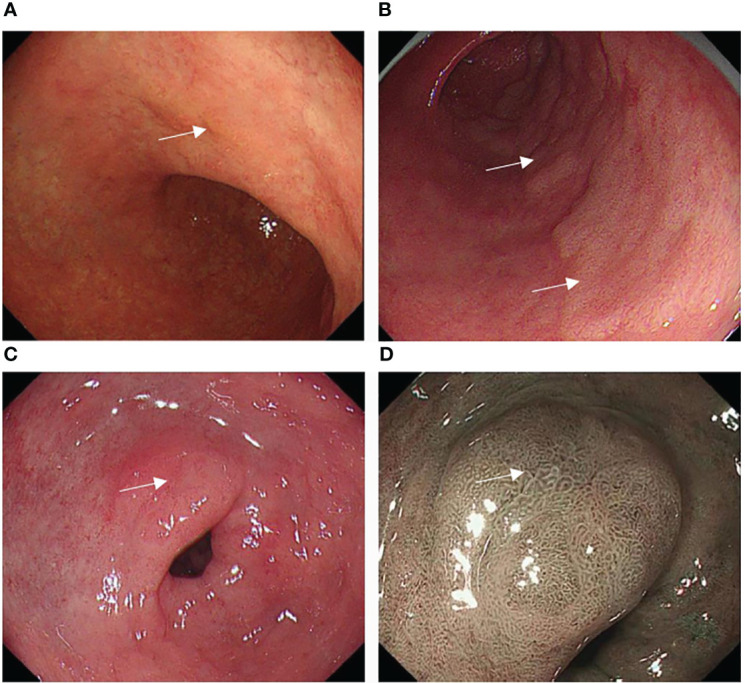
Typical color change and gastritis-like appearance of EGC after eradication: **(A)** A slightly depressed yellowish lesion on the small curved side of the corpus (white square). **(B)** A slightly raised whitish lesion on the posterior wall of the antrum (white square). **(C)** A slightly excavated reddish lesion on the small curved side of the antrum (white arrow), whose mucosa was similar to the surrounding non-cancerous area in WLE. **(D)** The lesion with a gastritis-like appearance presented regular microstructure (MS), microvascular (MV), and clear border in NBI-ME.

The tumor location, size, color, morphology, and gastritis-like appearance were observed in White Light Endoscopy (WLE). The tumor location referred to in the Japanese classification was divided into three portions: the upper (U), middle (M), and lower (L) parts (17). Tumor size was assessed at the longest diameter. Morphology was analyzed based on the Paris endoscopic classification (18). The tumor color was divided into three arms: yellowish, whitish, and reddish color. The color was defined as a more yellowish, whitish, or reddish color on most of the tumorous area rather than on the non-cancerous mucosal area around the tumor (19) (**Figures 2A–C**). The gastritis-like appearance was defined as a slight elevation or depression morphology in WLE, regular (open/closed-loop) microstructure (MS) mixed papillae or pits, and faint microvascular (MV) vessels in the narrow-band imaging with magnifying endoscopy (NBI-ME), with the mucosa not being significantly different from the surrounding non-cancerous area (12, 13) (**Figures 2C, D**).

The MS, MV, and border were shown in NBI-ME.

**Figure 3:**
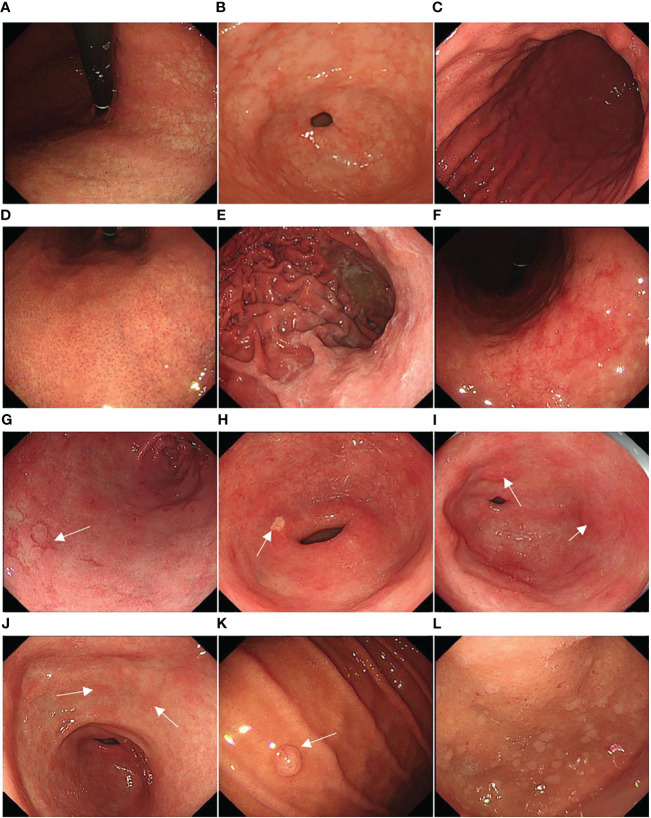
Representative background mucosa status: **(A)** gastric atrophy (GA), **(B)** intestinal metaplasia (IM), **(C)** diffuse redness (DR), **(D)** regular arrangement of collecting venules (RAC), **(E)** enlarged folds (EF), **(F)** map-like redness, **(G)** depressed erosion (white arrow), **(H)** xanthoma (white arrow), **(I)** raised erosion (white arrow), **(J)** patchy redness (white arrow), **(K)** fundic gland polyp (white arrow), and **(L)** multiple white and flat elevated lesions.

**Figure 4:**
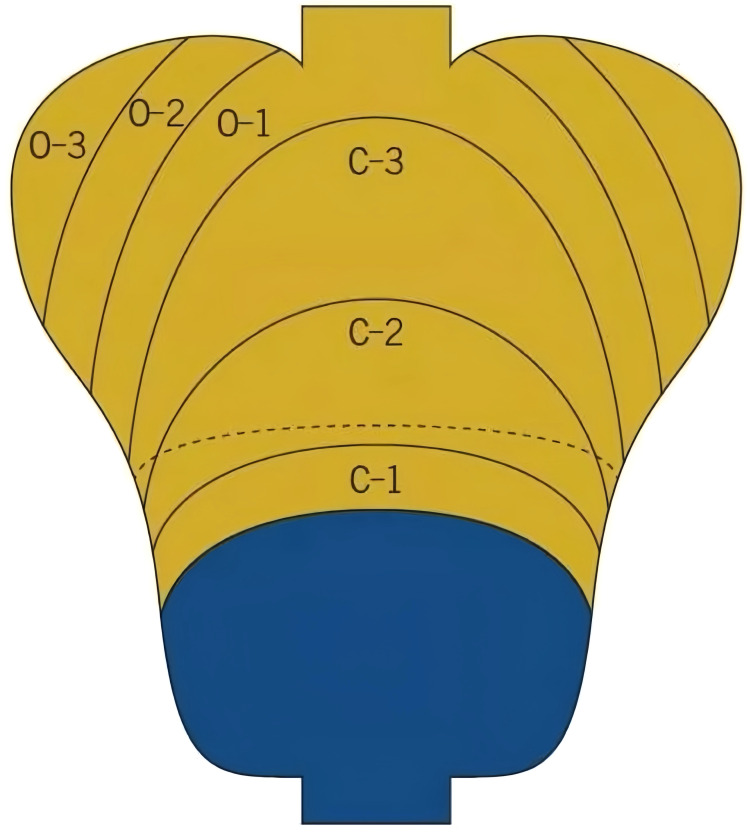
Kimura-Takamoto classification.

**Figure 5:**
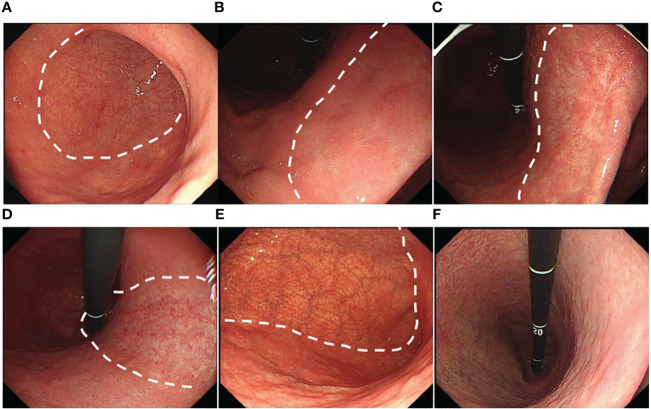
Grade of gastric atrophy (GA): **(A)** C1: GA limited to the antrum, **(B)** C2: GA limited to the antrum and lesser curvature of the distal gastric body, **(C)** C3: GA limited to the antrum and lesser curvature of the proximal gastric body, **(D)** O1: GA included the lesser curvature of the gastric body and the cardia, **(E)** O2: GA included the lesser curvature of the gastric body, anterior wall, and posterior wall, and **(F)** O3: GA widely included the whole gastric (GA border is indicated by a dotted line).

Background mucosa status was assessed according to the Kyoto gastritis classification (20), including GA, IM, DR, regular arrangement of collecting venules (RAC), EF, map-like redness, depressed erosion, xanthoma, raised erosion, patchy redness, fundic gland polyp, and multiple white and flat elevated lesions (**Figures 3A–L**). According to the location and extent of GA, the Kimura-Takemoto classification (**Figure 4**) was classified as closed (C-1, C-2, and C-3) and open (O-1, O-2, and O-3) (21) (**Figure 5**). The GA based on the Kimura-Takamoto classification was divided into a none/mild GA group (C0-C2), a moderate GA group (C3 and O1), and a severe GA group (O2 and O3) (22). There were three groups according to the extent of IM: IM group A (no IM), IM group B (IM in the antrum only), and IM group C (IM in the corpus only or in both the antrum and corpus) (22). DR contained three categories: none (the regular RAC was visible throughout the corpus), mild (the regular RAC was visible in part of the corpus), and severe (the absence of RAC). The EF was divided into two categories: none (the width of the folds was ≤4 mm under observation with a sufficient amount of air) and presence (the width of the folds was ≥5 mm). The others were classified as none and presence (13). The Kyoto gastritis scores were based on GA, IM, DR(RAC), and EF, and the sum of the five endoscopic findings was the Kyoto gastritis total score (**Table 1**) (20).

**Table 1:** Gastric cancer grade scores in the Kyoto Gastritis Classification.

Background		Score
GA	C0-C1	A0
	C2-C3	A1
	O1-O3	A2
IM	IM group A	IM0
	IM group B	IM1
	IM group C	IM2
DR(RAC)	none	DR0
	mild	DR1
	severe	DR2
EF	≤4mm	H0
	≥5mm	H1

GA, gastric atrophy; IM, intestinal metaplasia; DR, diffuse redness; RAC, regular arrangement of collecting venules; EF, enlarged folds.

### 2.6 The pathological data

The data included histological type, depth of invasion, vascular invasion, and Ki67 index. The histological type was divided into low-grade neoplasia, high-grade neoplasia, differentiated-type, and undifferentiated-type carcinoma, according to the Vienna classification (23). The depth of invasion was classified into two categories: M (tumor confined to mucosa) and SM (submucosal invasion) (17).

### 2.7 Statistical analysis

Statistical graphs were constructed to evaluate the trend of EGC. The continuous variables were calculated using the Mann-Whitney U-test and parametric data were expressed as the medians (range). The χ-squared test or Fisher’s exact test were applied for categorical variables that were presented in the table as the number of patients or lesions and their percentage. We compared the characteristics and background factors of EGC after eradication. In order to identify the independent background risks of EGC after eradication, the significant variables that were assumed to be univariate were put into a multivariable analysis using Logistic regression analysis. Meanwhile, we calculated the Kyoto gastritis total score in the EGC and non-EGC groups after eradication and speculated the risk of EGC after successful eradication.

Among the Hp-eradicated group, the background factors were also compared between IIc-type EGC and non-IIc-type EGC, to define the independent risk factors for IIc-type EGC. All statistical analyses were performed by SPSS statistical software and a p-value <0.05 was defined as having statistical significance.

## 3 Results

### 3.1 The trend of EGC after eradication

**Figure 6:**
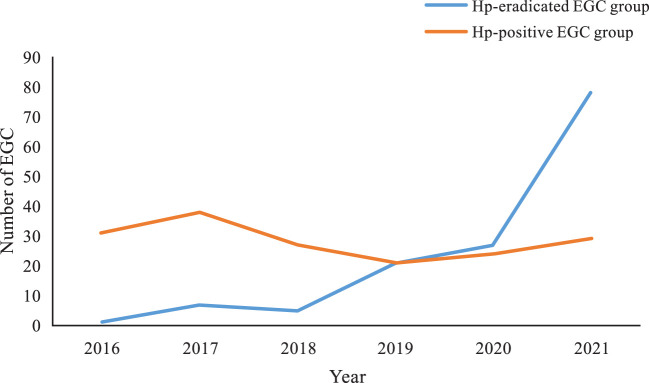
The distribution trend of Hp-eradicated and Hp-positive EGC groups between 2016 and 2021.

In the Hp-eradicated EGC group, the median duration after eradication was 18 months (range 13 to 38 months). The annual distribution of EGC after eradication varied greatly, with the number showing a significant upward trend. In 2016, there was only 1 lesion (0.7%) from 1 patient, whereas in 2017, there were 7 lesions (5%) out of 6 patients, and in 2018, there were 5 lesions (3.5%) from 5 patients. The number of EGCs has increased significantly since 2019: 21 lesions (15.1%) from 21 patients in 2019, 27 lesions (19.4%) out of 26 patients in 2020, with the number of EGCs peaking in 2021, with 78 lesions (56.1%) out of 74 patients (**Figure 6**). Compared with the Hp-eradicated EGC group, the Hp-positive EGC group showed a relatively flat downward trend. The number of EGCs that occurred in 2016 and 2017 was 29 patients (31 lesions) and 34 patients (38 lesions), respectively, with a total proportion of 40.5%. The number of EGCs decreased slightly after 2018, with 25 patients (27 lesions) in 2018, 20 patients (21 lesions) in 2019, 22 patients (24 lesions) in 2020, and 28 patients (29 lesions) in 2021 (**Figure 6**).

**Figure 7:**
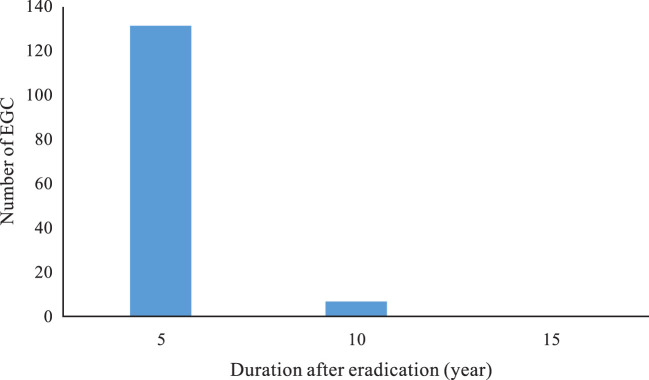
The distribution of EGC in duration after Hp eradication.

The 131 lesions (94%) out of 125 patients were found within 5 years after eradication, however, 7 lesions (5%) from 7 patients and 1 lesion (1%) from 1 patient were identified within 10 and 15 years, respectively, after successful eradication (**Figure 7**).

### 3.2 Clinical features of EGC after eradication

Female patients were more frequent in the Hp-eradicated EGC group than in the control group (p = 0.003). Patients in the eradication group were more likely to have a family history of gastric cancer (p = 0.047), however, the Hp-positive group was more likely to have a history of drinking (p = 0.009) and smoking (p = 0.004). There was no significant difference between the two groups in terms of age (**Table 2**).

**Table 2:** Comparison of clinical characteristics of HP-eradicated and HP-positive ECG groups.

		Hp-eradicated EGC group (n = 133), n (%)	Hp-positive EGC group (n = 158), n (%)	P-value
Sex	Female	58 (44)	43 (27)	0.003
	Male	75 (56)	115 (73)	
Median age (range) (year)		60 (59-61)	61 (59-62)	0.369
Family history of EGC	-	111 (83)	144 (91)	0.047
	+	22 (17)	14 (9)	
Drinking	-	100 (75)	96 (61)	0.009
	+	33 (25)	62 (39)	
Smoking	-	93 (70)	84 (53)	0.004
	+	40 (30)	74 (47)	

### 3.3 Endoscopic features of EGC after eradication

The lesions after eradication were more commonly located in the lower part of the stomach compared to those in the control group (p =0.001). Similarly, the yellowish color was more frequent in the eradicated group (p = 0.031). Tumor size was smaller in the eradicated group than in the positive group (p =0.001), with a median tumor size of 12mm (range 10 to 20mm). Although morphology was not statistically significant, the IIc-type EGC accounted for the largest proportion in the eradication group (60%). Meanwhile, the gastritis-like appearance was only found in the eradication group (p =0.001). The lesions with regular MS, MV, and indistinct border in the Hp-eradication group were more frequent than in the control group (P=0.008,0.003, 0.01, respectively) (**Table 3**).

**Table 3:** Comparison of endoscopic characteristics of HP-eradicated and HP-positive ECG groups.

		Hp-eradicated EGC group (n = 139), n (%)	Hp-positive EGC group (n = 170), n (%)	P-value
Tumor location	Upper	13 (9)	5 (3)	=0.001
	Middle	10 (7)	44 (26)	
	Lower	116 (84)	121 (71)	
Median tumor size (range) (mm)		12 (10-20)	15 (11-20)	=0.001
Tumor color	Reddish	73 (53)	104 (61)	0.031
	Yellowish	57 (41)	47 (28)	
	Whitish	9 (6)	19 (11)	
Morphology	IIa	42 (30)	43 (25)	0.912
	IIb	2 (2)	3 (2)	
	IIc	84 (60)	111 (65)	
	IIc+IIa	0	1 (0.5)	
	IIa+IIc	9 (7)	10 (6)	
	IIa+Ip	0 (0)	1 (0.5)	
	Is	1 (0.7)	1 (0.5)	
	Ip	1 (0.7)	0	
Gastritis-like appearance	None	115 (83)	170 (100)	=0.001
	Presence	24 (17)	0	
MS	Regular	7 (5)	0	0.008
	Irregular	129 (93)	167 (98)	
	None	3 (2)	3 (2)	
MV	Regular	6 (4)	0	0.003
	Irregular	132 (95)	170 (100)	
	None	1 (0.7)	0	
Border	Distinct	7 (5)	0	0.01
	Indistinct	132 (95)	170 (100)	

MS, microstructure; MV, microvascular.

### 3.4 Pathological features of EGC after eradication

There were no significant differences between the groups in terms of histological type, depth of invasion, vascular invasion, and Ki67 index. However, the Ki67 index was relatively lower in the eradication group, with the median Ki67 index of 45 (range 30 to 60) (**Table 4**).

**Table 4:** Comparison of pathological characteristics of HP-eradicated and HP-positive ECG groups.

		Hp-eradicated EGC group (n = 139), n (%)	Hp-positive EGC group (n = 170), n (%)	P-value
Histological type	Low-grade neoplasia	75 (54)	81(48)	0.439
	High-grade neoplasia	31 (22)	44 (26)	
	Differentiated-type carcinoma	26 (19)	40 (23)	
	Undifferentiated-type carcinoma	7 (5)	5 (3)	
Depth of invasion	Mucosa	135 (97)	166 (98)	1
	Submocosa	4 (3)	4 (2)	
Vascular invasion	–	132 (95)	168 (99)	0.096
	+	7 (5)	2 (1)	
Median Ki67 index (range)		45 (30-60)	50 (30-60)	0.329

### 3.5 Background mucosa status of EGC after eradication

Univariate analysis indicated that moderate/severe GA (C3-O3), IM group C (IM in the corpus only or in both the antrum and corpus), severe diffuse redness, and map-like redness were risk mucosal factors of EGC after eradication (P=0.001, =0.001, =0.001, 0.005, respectively) (**Table 5**). However, these were not independent risk factors for gastric cancer after eradication by logistic regression analysis (**Table 6**).

**Table 5:** Comparison of background mucosa status in the EGC group and the non-EGC group after eradication.

		Hp-eradicated EGC group (n = 133), n (%)	Hp-eradicated non-EGC group (n = 107), n (%)	P-value
GA	None/mild GA group	67 (50)	85 (79)	<0.001
	Moderate GA group	61 (46)	22 (21)	
	Severe GA group	5 (46)	0	
IM	IM group A	0	12 (11)	<0.001
	IM group B	35 (26)	34 (32)	
	IM group C	98 (74)	61 (57)	
DR	None	0	42 (39)	<0.001
	Mild	68 (51)	55 (51)	
	Severe	65 (49)	10 (10)	
EF	None	131 (99)	107 (100)	0.504
	Presence	2 (1)	0	
Map-like redness	None	78 (59)	81 (76)	0.005
	Presence	55 (41)	26 (24)	
Depressed erosion	None	97 (73)	85 (79)	0.24
	Presence	36 (27)	22 (21)	
Xanthoma	None	113 (85)	98 (92)	0.11
	Presence	20 (15)	9 (8)	
Raised erosion	None	114 (86)	82 (77)	0.071
	Presence	19 (14)	25 (23)	
Patchy redness	None	63 (47)	46 (43)	0.498
	Presence	70 (53)	61 (57)	
Fundic gland polyp	None	131 (99)	105 (99)	1
	Presence	2 (1)	2 (1)	
Multiple white and flat elevated lesions	None	129 (97)	104 (97)	1
	Presence	4 (3)	3 (3)	

GA, gastric atrophy; IM, intestinal metaplasia; DR, diffuse redness; EF, enlarged folds.

**Table 6:** Multivariate analysis of background in the EGC group and the non-EGC group after eradication.

		With/without ECG	Multivariate analysis		
			OR	95%CI	P-value
GA	None/mild GA group	71/89	1		
	Moderate GA group	59/18	1.86	0.84-4.09	0.121
	Severe GA group	9/0	-	-	0.999
IM	IM group A	0/12	1		
	IM group B	35/34	-	-	0.998
	IM group C	104/61	-	-	0.998
DR	None	0/42	1		
	Mild	70/55	-	-	0.997
	Severe	69/10	-	-	0.997
Map-like redness	None	58/26	1		
	Presence	81/81	1.21	0.55-2.64	0.632

GA, gastric atrophy; IM, intestinal metaplasia; DR, diffuse redness.

### 3.6 Kyoto gastritis score of EGC after eradication

The Kyoto gastritis total score of the EGC group was significantly higher than the non-EGC group (4 vs. 3, P <0.001). However, the four endoscopic findings were not significantly different between the two groups (**Table 7**).

**Table 7:** Comparison of Kyoto gastritis score in the EGC group and the non-EGC group after eradication.

	Hp-eradicated EGC group (n = 133)	Hp-eradicated non-EGC group (n = 107)	P-value
Kyoto gastritis total score	4 (3-5)	3 (1-4)	=0.001
GA	1 (0-1)	1 (0-1)	0.390
IM	2 (1-2)	2 (1-2)	0.410
DR	1 (1-2)	1 (0-1)	0.364
EF	0 (0-0)	0 (0-0)	0.204

GA, gastric atrophy; IM, intestinal metaplasia; DR, diffuse redness; EF, enlarged folds.

### 3.7 Correlation analysis of the IIc and non-IIc EGC in the background mucosal factors

The IIc-type EGC was the most common in the eradication group (60%) (**Table 3**), and the other types were uniformly classified as non-IIc EGC (40%). Among all background mucosa status, only moderate GA (C3-O1) (P=0.03) and depressed erosion (P=0.003) were risk factors. However, depressed erosion(OR=3.42, 95% CI: 1.35~8.65, P=0.009)only was an independent risk factor for IIc-type EGC in the multivariate analysis (**Table 8**).

**Table 8:** Association between the mucosa status and Macroscopic type (IIc and non-IIc EGC).

		IIc/ non-IIc	Univariate analysis			Multivariate analysis		
			OR	95%CI	P-value	OR	95%CI	P-value
GA	None/mild GA group	49/21		1				
	Moderate GA group	33/30	0.47	0.23-0.96	0.03	0.55	0.26-1.08	0.16
	Severe GA group	2/4	0.21	0.03-1.26	0.08	0.31	0.05-1.88	0.2
Depressed erosion	None	53/48		1				
	Presence	31/7	4.01	1.67-9.94	0.003	3.42	1.35-8.65	0.009

GA, gastric atrophy.

## 4 Discussion

In this study, the number of EGCs after eradication varied greatly in different years but overall showed an upward trend. However, the Hp-positive EGC group showed a relatively flat downward trend. The Kyoto Gastritis Consensus advocated that Hp gastritis should be defined as an infectious disease that is closely related to the development of gastric cancer, and all patients with Hp infection should receive eradication treatment (6). China, as a developing country, has a high prevalence of Hp infection rate and a high incidence of gastric cancer (24). The publication of the Kyoto Gastritis Consensus promoted the domestic standardization of Hp diagnosis and treatment and improved the awareness of Hp eradication. Meanwhile, patients with Hp infection in most parts of China have received standard eradication treatment. Thus, the population undergoing eradication has relatively increased. However, the effectiveness of Hp eradication for the prevention of gastric cancer depends on the severity of gastric mucosal damage (25), and some patients with GA, IM, and dysplasia who receive eradication treatment may be a potential population for gastric cancer after eradication. In this study, the increase in EGC numbers after eradication suggests that it should be a focus during endoscopy.

EGC can be detected within 15 years after eradication, but most cases were found within 5 years after eradication (131/139, 94%). A retrospective study found that the percentage of EGC detected gradually increased over time from successful eradication, especially in the fifth year after eradication, and the percentage of EGC detected was significantly higher than that in patients with infection (26). Take et al. found that the risk of developing gastric cancer in the second 10 years after eradication was significantly greater than in the first 10 years (27). Therefore, patients receiving successful eradication therapy should have regular endoscopic surveillance early within 5 years, and later follow-up should be extended to 10 or even 15 years. In addition, women and patients with a family history of gastric cancer were significantly higher in the eradication group than in the infection group. Therefore, these groups of patients after successful eradication should receive more focus on endoscopic screening. 

In the present study, EGC after eradication had a smaller tumor size, which was consistent with previous studies (8, 28, 29). The reports have found that gastric cancer detected after successful Hp eradication was located in middle and lower anatomical locations (8, 30). However, Horiguchi et al. (11) found that the prevalence of lesions located in the upper anatomical position in the eradication group was higher than that in the control group. Our study confirmed that lesions were mainly located in the lower part of the stomach in the eradication group when compared to the control group (84% versus 71%). We have also shown that yellowish appearance was one of the clinical features of EGC after eradication, something that was not indicated in the other articles (11, 19). Therefore, under WLE, yellowish and smaller lesions located in the lower site should be the focus for patients after Hp eradication.

In our study, the gastritis-like appearance (24 patients and 24 lesions) was only found in the HP-eradication EGC group. Meanwhile, regular MS, regular MV, and blurred borders were more common in the EGC group after eradication. Saka and Kobayashi et al. (12, 13) proposed that gastritis-like appearance was associated with surface differentiation and epithelium with low-grade atypia (ELA). The surface differentiation was defined as the absence of Ki-67 positive cells in the surface layer of the tumor (13), and the ELA was normal columnar epithelium appearing on the tumor surface (14). Then, the Ki-67 markers were not found by immunohistochemical staining in ELA (31). Some research papers have also found that the Ki-67 index was lower in the eradicated group than in the positive group (32, 33), which could confirm the findings of Saka and Kobayashi. Our study also found that the Ki-67 index was lower in the EGC group after eradication (45 versus 50) and the prevalence of low-grade neoplasia was higher in lesions with gastritis-like appearance (15/24, 63%), which could also confirm this theory, but the relationship between the two elements should be further explored in future studies. Meanwhile, relevant studies have shown that the submucosal invasion of gastric cancer is more frequent after eradication (8, 27). However, our result was the opposite, which may be due to the enrolled lesions being more low-grade (75/139, 54%) and high-grade neoplasia (31/139, 22%).

Previous reports described the features of gastric cancer after eradication as having a flat or depressed morphology (8, 14, 34), which may be related to the inhibition of proliferative ability after eradication (14, 28, 32, 33). The lower Ki-67 index in the EGC group from our study supports this because it was a key marker for promoting the proliferation of tumor cells (35, 36). Meanwhile, the prevalence of IIc-type EGC was also significantly higher in the HP-eradication EGC group in our study (84/139, 60%). We also found that depressed erosion was an independent risk factor for IIc-type EGC(OR=3.42, 95% CI: 1.35~8.65, P=0.009). Therefore, in cases of IIc-type EGC, clinicians should be vigilant after eradication, especially regarding the status of the depressed erosion background mucosa.

There are studies regarding risk factors of gastric cancer development after eradication. Toyoshima et al. (37) showed that severe GA was an independent risk factor for gastric cancer after eradication. Kodama et al. (38) confirmed that IM in the corpus was another risk factor. Moribata et al. (39) also proposed that map-like redness may be a positive predictor of gastric cancer after eradication. We found that moderate/severe GA, IM in the corpus, severe diffuse redness, and map-like redness were risk factors of EGC after eradication, and these will be key clues to evaluate the presence of gastric cancer after eradication. This result was compatible with the findings of Ohno et al. (40) based on the Kyoto classification of gastritis. However, the findings of the multivariate analysis did not obtain the same expected effect. Meanwhile, the Kyoto total risk score after eradication in the EGC group was 4, which was significantly higher than the non-EGC group. Toyoshima et al. (41) suggested that a Kyoto classification score of ≥ 4 in patients with Hp infection might indicate gastric cancer risk. Therefore, we speculate that those with a Kyoto risk score of 4 or higher may have a higher risk of gastric cancer.

Western and Japanese pathologists have different diagnostic criteria for dividing epithelial neoplasia and early gastric cancer. The invasion was the most important diagnostic criterion for most Western pathologists, whereas, for Japanese pathologists, nuclear features and glandular structures were more important (42). The Vienna classification classified low-grade and high-grade neoplasia as non-invasive carcinoma, solving differences in diagnostic criteria between Japanese and Western pathologists (23). At present, high-grade neoplasia could be considered as carcinoma (42). In this study, high-grade and low-grade neoplasia were included in the EGC group because they met the diagnostic criteria of EGC under endoscopic examination; in addition, we found obvious atypia of the nucleus and glandular structures in pathology.

Based on our findings, each patient after Hp eradication should be risk-stratified in terms of clinical, pathological, endoscopic characteristics and background factors to establish an individualized endoscopic surveillance strategy for at least 15 years. There are several limitations in the present study. First, our study subjects included low-grade and high-grade neoplasia. Thus, our results might not represent the characteristics of all gastric cancers after eradication. Second, the enrolled groups were screened by telephone follow-up, and dates were collected through search, which might have certain retrospective selection biases. Third, the differences in pictures preserved in the system may have interfered with the assessments.

In conclusion, the endoscopic features of EGC after eradication are smaller, yellowish, and located in the lower part of the stomach. The risk factors include moderate/severe GA, IM in the corpus, severe diffuse redness, and map-like redness. The Kyoto classification total score of 4 or more after successful eradication treatment might indicate EGC risk. In addition, in cases of depressed erosion after eradication, there should be an awareness of the risk of IIc-type EGC.

## Data availability statement

The raw data supporting the conclusions of this article will be made available by the authors, without undue reservation.

## Ethics statement

The studies involving humans were approved by The Ethics Committee of the Affiliated Hospital of Qingdao University (ethics batch number QYFY WZLL 27484). The studies were conducted in accordance with the local legislation and institutional requirements. Written informed consent for participation was not required from the participants or the participants’ legal guardians/next of kin in accordance with the national legislation and institutional requirements. Written informed consent was obtained from the individual(s) for the publication of any potentially identifiable images or data included in this article.

## Author contributions

YW: Data curation, Investigation, Methodology, Validation, Writing – original draft. CM: Supervision, Validation, Writing – review & editing. CZ: Writing – review & editing. YL: Writing – original draft. XW: Writing – review & editing. XJ: Writing – review & editing. YY: Writing – review & editing. XL: Writing – review & editing. XY: Conceptualization, Data curation, Investigation, Resources, Supervision, Visualization, Writing – review & editing.
